# The impact of LCTI on China's low-carbon transformation from the spatial spillover perspective

**DOI:** 10.1371/journal.pone.0242425

**Published:** 2020-11-23

**Authors:** Wenchao Li, Jian Xu, Zhengming Wang, Jialiang Yang

**Affiliations:** School of Finance and Economics, Jiangsu University, Zhenjiang City, Jiangsu Province, China; China University of Mining and Technology, CHINA

## Abstract

China has conducted a long-term low-carbon technology innovation (LCTI), but there was a faster increase of CO_2_ emission in 2017 and 2018 than in 2016, which has lead scholars to doubt the effect of LCTI on CO_2_ emission. This paper builds a spatial auto regression (SAR) model with provincial panel data from 2011 to 2017 to calculate the spatial spillover effect of China's LCTI on regional CO_2_ emission. The results show that regional LCTI can reduce the local CO_2_ emission, but will increase the CO_2_ emission of adjacent regions due to spatial spillover effect. This produces the uncertainty of the promotion effect of LCTI on China's low-carbon transformation. Therefore, this paper suggests innovation resources should be appropriately and evenly distributed among regions to avoid their agglomeration in few regions.

## Introduction

**Fig 1:**
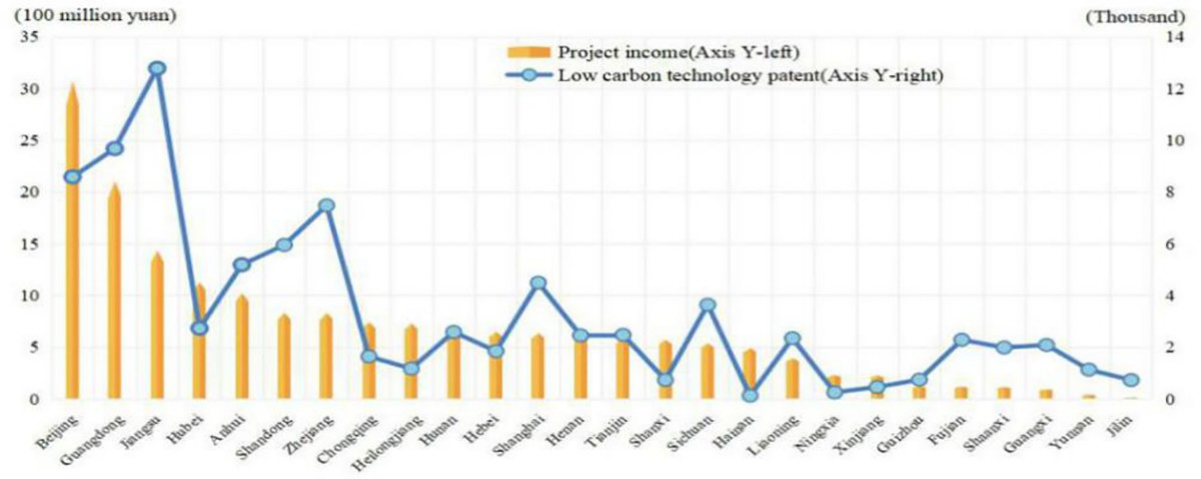
Regional low-carbon technology patents and environmental project income. Data sources: Tonghuashun database (http://www.51ifind.com/ Accessed 14 March 2020) & incopat database (https://www.incopat.com/ Accessed 13 March 2020.). Notice: there are only 26 regions, as five other regions lacking the project income data.

There will be only ten years left for China to honor the pledge to peak its emissions by 2030. Low-carbon technology innovation is highly anticipated in China, where many policies have been carried out to promote low-carbon technology innovation (LCTI). Technological patents are the core of LCTI, while low-carbon project revenue reflects the commercial value of the technology innovation. Recently, low-carbon technology patents and the low-carbon project revenue (environmental project) in China have increased a lot (Fig 1), especially in the field of new energy power generation, new energy vehicle, green sharing technology and etc.

As shown in Fig 1, a few regions generally have more innovations than other regions. Specifically, Beijing, Guangdong and Jiangsu are taking the lead in terms of the numbers of patents and revenue. Despite the increasing innovation, the growth of China's CO_2_ emission is higher in 2017 and 2018 than in 2016. Therefore, this article mainly focuses on the counterintuitive relations between LCTI and CO_2_ emission. The research conclusion shows that the spatial spillover effect of LCTI in adjacent regions increases the carbon emission in this region, leading to ineffective curb on China's carbon emission. Based on this, relevant policy suggestions are proposed for the development of China's low-carbon technology innovation.

## Literature review

In China, the national and some regional carbon emission is decreasing [1]. However, there is altogether a large amount of CO_2_ emission in China, most of which is from traditional industries [2]. To achieve the emission peak in 2030, China needs to reduce 12.5Gt CO_2_ emission relative to 2015 [3].

Previous studies have proposed various suggestions on carbon emission reduction from different perspectives. From the perspective of energy consumption, low-carbon transformation can be realized by reducing the proportion of coal consumption and increasing the proportion of natural gas and renewable energy consumption [4]. From the perspective of industrial structure, developing low-carbon industries [5] and eliminating enterprises with high energy consumption in traditional industries [6] are the main paths to control CO_2_ emission. From the perspective of market trading, the construction of the carbon trading market can achieve a win-win situation of economic growth and low-carbon transformation [7]. From the perspective of environmental regulation, imposing heavy taxes on enterprises with high-energy consumption can make them exit the market [8].

All the literature conclusions above admite that the technology innovation is important to reduce CO_2_ emission: without economic alternative technology, it is difficult to reduce the proportion of coal consumption. Without the core technologies of the low-carbon industry, it is difficult to develop low-carbon industries and reduce backward production capacity. Without carbon productivity improvement, it is difficult for the carbon quota seller to make profits. Without specific low-carbon technology for traditional industries, environmental regulation can only restrict economic development rather than achieve low-carbon transformation.

**Fig 2:**
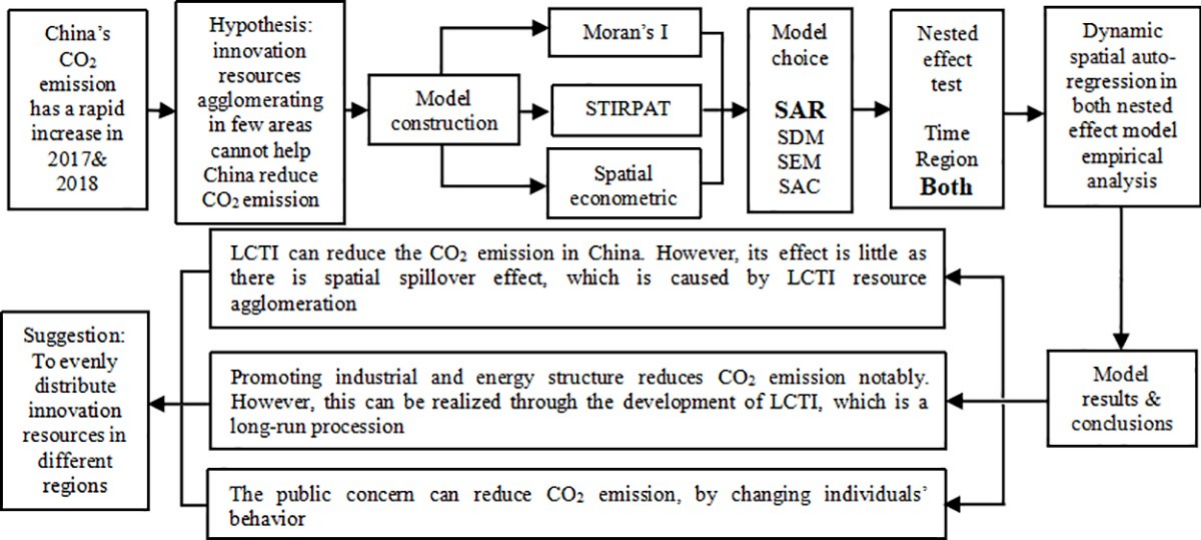
The research framework diagram.

Once a low-carbon technology patent has commercial value, it will impose effects on the low-carbon transformation with other factors such as the industrial structure, per capital GDP, energy structure, foreign investment, public concern and etc. [9–17]. However, such a development mode is controversial [18]. Although it forms the agglomeration of low-carbon technology innovation resources and accelerates the low-carbon transformation [19], it leads to an unbalanced allocation of low-carbon technology innovation resources [20]. The article proposes the hypothesis that the regional agglomeration effect of LCTI offsets its inhibition effect on CO_2_ emission, that is, the spatial spillover effect of LCTI on CO_2_ emission is positive. It is because the innovation resources of low-carbon technology are transferred to the agglomeration region, making adjacent regions unable to make full use of the innovation resources to reduce their CO_2_ emission. If the positive spillover effect is significant, China's low-carbon transformation may be restrained. On this basis, the article conducts an empirical analysis to verify the above hypothesis. The research framework is as follows (Fig 2).

### Methodology

To explore whether there exists the LCTI spatial spillover effect on CO_2_ emission, the article should test whether there are spatial autocorrelation in LCTI and CO_2_ emission. Therefore, the article calculates the Moran’s Index to test whether there is spatial autocorrelation between LCTI and CO_2_ emission at first, and then the spatial econometric model about LCTI spatial spillover effect on CO_2_ emission is constructed based on Stochastic Impacts by Regression method on Population, Affluence, and Technology (STIRPAT). To avoid the mismatch between the geographical and economical contiguity, the article chooses economic spatial weight matrix (Eq 1) to calculate the Moran’s Index.

$$\left\{ \begin{array}{l} \bar{Y}=\frac{\sum_{i=1}^{n}\sum_{t=t_{0}}^{t_{1}}Y_{i,t}}{n(t_{1}-t_{0}+1)};\bar{Y_{i}}=\frac{1}{(t_{1}-t_{0}+1)\sum_{t=t_{0}}^{t_{1}}Y_{i,t}}\\ w=w_{i}diag(\frac{\bar{Y_{1}}}{\bar{Y}},\frac{\bar{Y_{2}}}{\bar{Y}},\cdots ,\frac{\bar{Y_{n}}}{\bar{Y}}) \end{array}\right.$$ (1)

In Eq 1, *w* is the spatial weight matrix, *w*_*i*_ is the spatial weight matrix of geographic distance, and $\bar{Y_{i}}$ is the weighted average GDP of the region (*i*). *t*_0_ & *t*_1_ is the initial year and the end year of the sample, and $\bar{Y}$ is the weighted average GDP of all regions. Furthermore, the Moran’s Index (*Moran*'*sI*) is measured, and the specific formula is shown in Eq 2:

$$Moran'sI=\frac{\sum_{i=1}^{n}\sum_{c=1}^{n}w_{i,c}(CE_{i}-\bar{CE})(CE_{c}-\bar{CE})}{S^{2}\sum_{i=1}^{n}\sum_{c=1}^{n}w_{i,c}},Moran'sI\in [-1,1]$$ (2)

In Eq 2, *CE*_*i*_ & *CE*_*c*_ represent the CO_2_ emission of region *i* & *c*, *ω*_*ic*_ is the spatial weight matrix, which shows regional and correlation characteristics between the regions *i* & *c*, and $\bar{CE}$ is the average CO_2_ emission in all the regions. In Eq 2, *S* represents the geographic coordinate distance between capitals of region *i* and *c* (this article regards municipality itself as the provincial capital). For calculating the LCTI Moran’s Index, *CE* should be substituted with *LP* (low-carbon patent). The spatial agglomeration is stronger when the Moran’s Index is closer to 1. Moran’s Index reveals the global spatial autocorrelation, and exponential scatter diagram reveals local spatial autocorrelation. If the spatial autocorrelation exists, the article will construct the basic panel data model in nested effect (Eq 3).

$$\begin{array}{l} \ln CE_{it}=\beta_{0}+\beta_{1}\ln LP_{it}+\beta_{2}\ln IS_{it}+\beta_{3}\ln GDP_{it}+\beta_{4}\ln ES_{it}+\beta_{5}\ln FI_{it}+\\ \beta_{6}\ln PC_{it}+\beta_{7}\ln EGI_{it}+\beta_{8}\ln SC_{it}+\lambda_{t}+\mu_{\varphi }+\varepsilon_{it} \end{array}$$ (3)

In Eq 3, *i* represents the region, *t* represent the time, and *CE* represents the CO_2_ emission. The core explanatory variable *LP* represents LCTI level. The other explanatory variables are industrial structure (*IS*), regional per capita GDP (*GDP*), energy structure (*ES*), foreign investment (*FI*), public concern index of environmental pollution (*PC*), environmental government invest per GDP (*EGI*), and regional sewage charges (*SC*). Meanwhile, the time nested variable is *λ*_*t*_, the regional nested variable is *μ*_*φ*_, and error is *ε*_*it*_. At last, this article can construct the spatial panel model (Eq 4) based on Eq 3.

$$\left\{ \begin{array}{l} CE_{it}=\tau CE_{i,t-1}+\rho \omega_{i}^{'}CE_{t}+x_{it}^{'}\beta +d_{i}^{'}X_{i}\delta +\lambda_{t}+\mu_{\varphi }+\varepsilon_{it}\\ x_{it}^{'}\beta =\beta_{1}\ln LP_{it}+\beta_{2}\ln IS_{it}+\beta_{3}\ln GDP_{it}+\beta_{4}\ln ES_{it}+\beta_{5}\ln FI_{it}+\\ \beta_{6}\ln PC_{it}+\beta_{7}\ln EGI_{it}+\beta_{8}\ln SC_{it}\\ \varepsilon_{it}=\lambda m_{i}^{'}\varepsilon_{i}+v_{it} \end{array}\right.$$ (4)

In Eq 4, the term $d_{i}^{'}X_{i}\delta$ is the explanatory variable of the spatial lag effect, and the term $\rho \omega_{i}^{'}CE_{t}$ is the explained variable of the spatial lag effect of. $d_{i}^{'}$, $\omega_{i}^{'}$ and $m_{i}^{'}$ represent the line *i* in spatial weighted matrix of explanatory variable, explained variable, and perturbation term. The variables *τ*, *ρ*, *δ*, and *λ* are the determination coefficients: If *λ* = 0, Eq 4 is a Spatial Durbin Model; If *λ* = 0 & *δ* = 0, Eq 4 is a Spatial Autoregressive Model; If *τ* = 0 & *δ* = 0, Eq 4 is a Spatial Autocorrelation Model; If *τ* = *ρ* = 0 & *δ* = 0, Eq 4 is a Spatial Error Model.

### Ethics statement

The data of this paper comes from authoritative data sets such as China Statistical Yearbook and China logistics yearbook, which can be found on the open website.

## Variables and data

### Core variables

There are two core variables in this article: CO_2_ emission and LCTI. As the team of China Emission Accounts and Datasets (CEADs) calculated the CO_2_ emission of China from 17 kinds of energy and 47 industries, so the article uses the CEADs’ CO_2_ emission data of China [20].

The article uses the number of technology patents classified by Y02 in the Cooperative Patent Classification (CPC) published in the incopat database in October 2017 to represent the level of LCTI (Table 1). The retrieval scope covers all the patents applied in China from 2011 to 2017, and the retrieval formula is ((CPC = (Y02) AND (AP-COUNTRY = (CN)) AND (AD = 2011)).

**Table 1:** The classification of low-carbon technology patents.

Codes	Name	Codes	Name
Y02B	Building-related low-carbon technologies	Y02T	Transportation-related low-carbon technologies
Y02C	Technologies of the capture, storage, storage or disposal of greenhouse gases	Y02W	Low-carbon technologies related to wastewater treatment or waste management
Y02E	Low-carbon technologies of energy generation, transmission and distribution	Y02P	Low-carbon technologies of goods production and processing of goods

### Other variables

In addition to LCTI, CO_2_ emission is also affected by other factors. Based on the existing research results, the article also considered the following explanatory variables: (1) Environmental Regulation. Studies have shown that under the heterogeneous environmental regulations, the agglomeration scale and CO_2_ emission of various manufacturing sectors present an inverted U-shaped trend [21]. This article measures the environmental regulation intensity of each region from three dimensions: government, enterprise, and public. The proportion of environmental pollution control investment to GDP represents the intensity of environmental regulation from the government. The total amount of pollutant charges in each region represents the intensity of environmental regulation from the enterprise. The index of public attention to environmental pollution represents the intensity of environmental regulation from residents. (2) Industrial Structure. Low-carbon industrial structure transformation can help build a sustainable industrial system, which can solve the problems of environmental pollution and economic slowdown [22, 23]. The article uses the proportion of the secondary industry to GDP to represent the CO_2_ emission status of the industrial structure in each region. (3) Economic Development and Market Opening. In addition to technological progress and industrial structure, economic development & foreign investment are the main factors affecting carbon emissions [24, 25], because CO_2_ emission in most regions of China has not decoupled with economic growth and foreign investment [26]. The article uses per capita GDP and foreign direct investment to represent economic development and market opening. (4) Energy Structure. The optimization of energy consumption structure is the main way to reduce carbon emission intensity [27]. The article uses the ratio of renewable energy consumption to total energy consumption to represent the energy structure.

### Data

The research period of the article is 2011–2017, with 30 regions (except Tibet) involved. The low-carbon patent data of each region was from the incopat patent database. CO_2_ emission data was from CEADs. The proportion of the secondary industry, foreign direct investment and per capita GDP data were from <China Statistical Yearbook>. The data on energy structure was from <China Energy Statistical Yearbook>. The data on the investment in environmental pollution control was from <China Environmental Yearbook>. The data of sewage charges were from <China Environmental Yearbook> and some regional environmental yearbooks [28]. The data of public concern on environmental pollution were from the official website of Baidu Index [29].

## Empirical analysis

### Spatial autocorrelation

**Fig 3:**
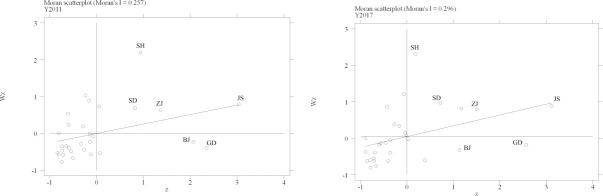
The Moran’s Index scatter diagram of LCTI in 2011 and 2017.

**Fig 4:**
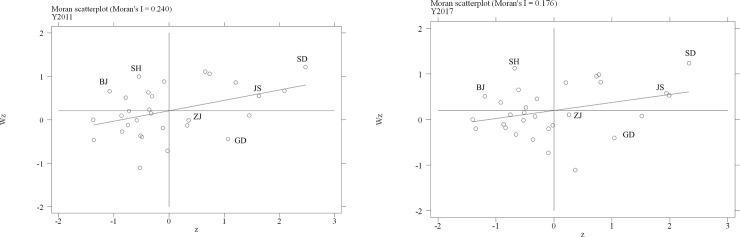
The Moran’s Index scatter diagram of CO_2_ emission in 2011 and 2017.

To measure the spatial autocorrelation between LCTI and CO_2_ emission, the article calculates the global Moran’s Index (Table 2) and draws Moran’s Index scatter diagram of both LCTI (Fig 3) and CO_2_ emission (Fig 4) in 30 regions.

**Table 2:** The global Moran’s Index of LCTI and CO_2_ emission from 2011 to 2017.

LCTI	2011	2012	2013	2014	2015	2016	2017
Moran’s I	0.257	0.287	0.278	0.286	0.331	0.327	0.296
Z-value	2.590	2.807	2.698	2.772	3.111	3.113	2.963
P-value	0.005	0.003	0.003	0.003	0.001	0.001	0.002
CO_2_ emission	2011	2012	2013	2014	2015	2016	2017
Moran’s I	0.240	0.232	0.224	0.225	0.217	0.206	0.176
Z-value	2.338	2.219	2.139	2.148	2.091	1.998	1.785
P-value	0.010	0.013	0.016	0.016	0.018	0.023	0.037

The global Moran’s Index of both LCTI and CO_2_ emission are positive (Table 2), which means there are spatial autocorrelation in both LCTI and CO2 emission. In Fig 3, most regions are agglomerating in the third quadrant. It shows that although the agglomeration of LCTI is comparatively high, the quality of agglomeration is low. And a few regions have a better LCTI agglomeration, such as Jiangsu (JS), Shandong (SD), Zhejiang (ZJ), and Shanghai (SH). In Fig 4, most regions agglomerate in the first and the third quadrant, with high-emission regions in the first quadrant and low-emission regions in the third quadrant. Meanwhile, there are some differences in few regions. Zhejiang (ZJ) and Guangdong (GD) can help neighbor regions reduce CO_2_ emission, whereas Beijing (BJ) and Shanghai (SH) increase neighbor regions’ CO_2_ emission. On this basis, a spatial econometric model is set up. There are four kinds of spatial econometric models: Spatial Autoregressive Model (SAR), Spatial Error Model (SEM), Spatial Durbin Model (SDM), and Spatial Autocorrelation Model (SAC). The article compares Log-likelihood Index, Moran's Index, Lagrange Multiplier, and Robust Lagrange Multiplier of these four models by model error test (Table 3). In Table 3, compared with other three models, the SAR model can be chosen for better coefficients.

**Table 3:** Results of spatial econometric model error test.

	SAR (dynamic)	SEM (static)	SDM (dynamic)	SAC (static)
Log-likelihood	323.43	312.12	232.45	310.13
Moran's Index	none	43.53*** (0.000)	none	none
Lagrange multiplier	25.47*** (0.001)	1.98 (0.096)	none	none
Robust Lagrange multiplier	23.75*** (0.000)	0.753 (0.271)	none	none

Notice: data in the bracket is P-value.

### Spillover effect of LCTI on carbon emissions

The article uses the maximum likelihood regression to calculate the LCTI’s impact on CO_2_ emission of China from 2011 to 2017. As the nested effect has a better result than the random effect in the Hausman test, the article chooses the nested effect SAR model to run the data. There are three kinds of nested effect model: time nested, region nested, and both nested, and the article should choose one of them by comparing their fitting results (Table 4). In Table 4, the results of the basic panel model in the nested effect and static SAR model are compared with the results of the dynamic SAR model.

**Table 4:** SAR model results of time-space spillover effect.

Variables	Basic panel model in nested effect	Static SAR model	Dynamic SAR model in time nested effect	Dynamic SAR model in region nested effect	Dynamic SAR model in both nested effect
LnCE_t-1_			1.055*** (0.013)	0.706*** (0.047)	0.693*** (0.048)
w*lnCE		0.052 (0.240)	0.295*** (0.040)	0.317** (0.151)	0.445* (0.268)
lnLP	-0.109*** (0.026)	0.110*** (0.026)	-0.012 (0.011)	-0.015 (0.020)	-0.003*** (0.001)
lnIS	0.118 (0.081)	0.118 (0.079)	0.043* (0.024)	0.127** (0.057)	0.183** (0.086)
lnGDP	-0.161* (0.087)	-0.160* (0.085)	0.039** (0.017)	0.033 (0.057)	-0.026 (0.067)
lnES	0.188** (0.082)	0.188** (0.081)	0.009 (0.018)	-0.063 (0.060)	-0.114* (0.064)
lnFI	0.036 (0.025)	0.036 (0.024)	-0.004 (0.009)	0.051*** (0.015)	0.028 (0.018)
lnPC	0.002 (0.010)	0.002 (0.010)	-0.023** (0.011)	-0.021* (0.012)	-0.030 (0.022)
lnEGI	0.028 (0.018)	0.028 (0.018)	0.018* (0.010)	-0.013 (0.013)	-0.011 (0.012)
lnSC	-0.015 (0.015)	-0.015 (0.024)	-0.023** (0.010)	-0.013 (0.009)	-0.011 (0.000)
error		0.004*** (0.000)	0.003*** (0.000)	0.002*** (0.000)	0.001*** (0.000)
Log-likelihood		297.232	279.727	330.928	281.122
R-sq	0.294	0.293	0.998	0.985	0.983
consant	4.594*** (0.465)				
Obs	210	180	180	180	180

Notice

* p<0.1

** p<0.05

*** p<0.01, and data in the bracket is standard error.

In Table 4, In the dynamic SAR model, the coefficient of the lag term of lnCE is positive, and are significant at 1% level, indicating that China's inter-provincial carbon dioxide emissions have a strong cumulative effect. SAR model can not only verify the spatial correlation of carbon dioxide emissions among provinces, making the estimation of the model more reliable, but also estimate the spillover effect of province-internal LCTI (direct effect), inter-provincial LCTI and overall LCTI (total effect) on China's carbon dioxide emissions respectively.

The w*lnCE coefficient is positive in the dynamic SAR model, which proves the positive spillover effect of the neighbor region on the local region. As a result, the dynamic SAR model in the both nested effect has better fitting coefficients (see p-value in the bracket). However, whether the nested effect exists depends on the both nested effect test (Table 5).

**Table 5:** The both nested effect test of the SAR model.

Likelihood-ratio test	LR chi2(10) = 19.68
(Assumption: region nested in both)	Prob > chi2 = 0.0003
Likelihood-ratio test	LR chi2(10) = 879.63
(Assumption: time nested in both)	Prob > chi2 = 0.0000

In Table 5, the both nested effect exists, and the explanatory variables of the local region (Local effect) and neighbor region (Spillover effect) jointly affect the local region’s carbon emissions. As explanatory variables are hard to change in the short term, the article makes regression analysis in the short term and long term separately (Table 6).

**Table 6:** The spatial effect of explanatory variables on CO_2_ emission.

Explanatory variables	Short term			Long term		
	Local effect	Spillover effect	Total effect	Local effect	Spillover effect	Total effect
lnLP	-0.004*** (0.007)	0.001*** (0.000)	-0.003*** (0.008)	-0.020*** (0.004)	0.011*** (0.003)	-0.009*** (0.001)
lnIS	0.194** (0.083)	-0.055** (0.024)	0.139** (0.069)	0.666*** (0.023)	-0.316** (0.095)	0.350** (0.092)
lnGDP	-0.030*** (0.002)	0.008** (0.003)	-0.022** (0.010)	-0.110* (0.061)	0.059** (0.021)	-0.050* (0.031)
lnES	-0.116* (0.062)	0.032** (0.011)	-0.084* (0.050)	-0.388** (0.101)	0.179** (0.069)	-0.209** (0.102)
lnFI	0.029* (0.005)	-0.008 (0.007)	0.021 (0.015)	0.101* (0.060)	-0.043 (0.123)	0.058 (0.130)
lnPC	-0.030** (0.012)	0.009*** (0.001)	-0.021*** (0.009)	-0.108*** (0.005)	0.059*** (0.003)	-0.049 *** (0.003)
lnEGI	-0.012** (0.006)	0.003** (0.001)	-0.008* (0.005)	-0.040 (0.061)	0.020 (0.077)	-0.020 (0.066)
lnSC	-0.010*** (0.002)	0.003** (0.001)	-0.007*** (0.001)	-0.036** (0.031)	0.017 (0.039)	-0.019 (0.038)

Notice

* p<0.1

** p<0.05

*** p<0.01, and data in the bracket is standard error.

In Table 6, the total effect of LCTI on CO_2_ emission is -0.003 in the short term and -0.009 in the long term. This means the LCTI can reduce the CO_2_ emission, and with the promotion of the industrial & energy structure, the reduction effect will be increased. However, compared with other explanatory variables, the reduction effect of LCTI is uncertain. Meanwhile, the spillover effect of LCTI on CO_2_ emission is 0.001 in the short term and 0.011 in the long term. It shows that the LCTI in neighbor regions increases the CO_2_ emission in the local region, especially in the long run. The results proved the assumption of the article: the CO_2_ emission in local region, will increase with the development of LCTI in neighbor regions.

Besides LCTI, there are other explanatory variables, and the analysis results of these explanatory variables are similar to those reported in the literature in Part 1, so the article will not repeat them here. However, the industrial (IS) and energy structure (ES) have a strong total effect on CO_2_ emission. This is because considering stability, safety and economy, energy consumption relies on fossil energy, especially the manufactory industry. Moreover, LCTI can promote the industrial & energy structure as reported in literature review. Meanwhile, the public concern about environmental pollution (PC) has a certain inhibitory effect on carbon emissions.

## Conclusions and suggestions

The article tests the impact of low-carbon technology innovation on CO_2_ emission by building a dynamic spatial auto-regression model with the panel data of 30 regions in China from 2011 to 2017. Conclusions and suggestions are as follows:

1. Low-carbon technology innovation can curb regional CO_2_ emissions, but its overall effect is weak at the national level. The regions with a good innovation environment attract low-carbon technological innovation resources from neighboring regions, which will lead to the improvement of low-carbon technological innovation in this region but increase CO_2_ emission in neighboring regions for lack of innovation resources. This indicates that the gathering of technology innovation resources can promote the rapid development of low-carbon technology innovation in a few regions at the beginning. However, at a certain stage of development, resources are excessively concentrated in a few regions, which slow down the innovation and development of low-carbon technologies in many other regions. Consequently, the transition of China's low-carbon economy will slow down and the CO_2_ emission will increase (e.g. in 2017 and 2018).
2. In the long run, the optimization of industrial and energy structure has an important impact on curbing CO_2_ emission. However, it is difficult to optimize the industrial and energy structure in the short term significantly without low-carbon technological innovations.
3. The residents’ awareness of a low-carbon life has an impact on CO_2_ emission. The awareness can promote the low-carbon transformation by changing the consciousness of the public, such as changing their consumption behavior, motivating them to prevent the emission from enterprises and propagandize the importance of low-carbon development to the society.
4. Based on the above conclusions, the article proposes that innovation resources should be appropriately and evenly distributed among regions to guide the transfer of talents and capital from the regions with abundant resources to other regions. The publicity and education should be intensified to call on the public to pay more attention to the ecological environment so that the public's low-carbon awareness will be improved and can play a greater role in the economic low-carbon transformation.
5. The government should strengthen and improve the democratic system in all aspects, increase the channels for citizens' environmental supervision, and improve relevant laws and regulations to protect citizens' environmental demands and environmental rights and interests. By improving the credibility of the government, the public will pay more attention to carbon emissions and participate in environmental governance, and the goal of carbon dioxide emission reduction can be achieved.

According to the results above, the optimization of industrial and energy structure has an important impact on curbing CO_2_ emission. In the literature review, we show that the LCTI can promote the industrial and energy structure. However, this paper has a limitation in data collection (at least 15–20 years’ data is needed, but the low-carbon data starts from 2011) to prove the mechanism. Therefore, the next step is to prove the mechanism that LCTI can promote the industrial and energy structure transition by collecting enough data.

### Limitation, and future work

However, this paper has some limitations in data selection and classification. Due to the availability of data, this paper takes the provincial area as the research object, which makes the amount of data relatively limited.

How to use low-carbon technology innovation to drive the low-carbon transformation of industrial and energy structure is a key point that needs further research.

## Supporting information

S1 Table The data of all variables. (DOCX)
