# Supplementary material for: The impact of LCTI on China's low-carbon transformation from the spatial spillover perspective
Source: PLoS One. 2020 Nov 23;15(11):e0242425. doi: 10.1371/journal.pone.0242425 (PMC7682850; doi:10.1371/journal.pone.0242425)
Supplement: S1 Table — (DOCX) [file pone.0242425.s001.docx]

| YEAR | AREA | CO2 | CE | IS | GDP | ES | FI | PC | EGI | SC |
| --- | --- | --- | --- | --- | --- | --- | --- | --- | --- | --- |
| 2011 | BeiJing | 94 | 6692 | 23 | 8.17 | 0.150 | 8896.93 | 5744 | 1.31 | 0.32 |
| 2011 | TianJin | 152 | 1581 | 52 | 8.52 | 0.118 | 7601.90 | 1458 | 1.55 | 2.26 |
| 2011 | HeBei | 725 | 1292 | 54 | 3.40 | 0.124 | 3026.01 | 5612 | 2.54 | 14.94 |
| 2011 | ShanXi | 439 | 462 | 59 | 3.14 | 0.111 | 2112.17 | 4187 | 2.21 | 19.95 |
| 2011 | NeiMengGu | 598 | 264 | 56 | 5.80 | 0.120 | 1689.74 | 2379 | 2.76 | 11.59 |
| 2011 | LiaoNing | 455 | 2115 | 55 | 5.08 | 0.101 | 10989.65 | 5400 | 1.69 | 14.47 |
| 2011 | JiLin | 234 | 585 | 53 | 3.85 | 0.085 | 1539.70 | 2306 | 0.96 | 3.70 |
| 2011 | HeiLongJiang | 247 | 790 | 50 | 3.28 | 0.083 | 1386.62 | 3009 | 1.21 | 4.58 |
| 2011 | ShangHai | 200 | 4096 | 41 | 8.26 | 0.146 | 24986.43 | 2763 | 0.75 | 2.36 |
| 2011 | JiangSu | 633 | 8915 | 51 | 6.23 | 0.191 | 37931.32 | 9995 | 1.17 | 20.26 |
| 2011 | ZheJiang | 379 | 5082 | 51 | 5.92 | 0.215 | 13370.06 | 7647 | 0.74 | 10.08 |
| 2011 | AnHui | 291 | 2073 | 54 | 2.57 | 0.142 | 2177.42 | 5784 | 1.75 | 4.92 |
| 2011 | FuJian | 237 | 1405 | 52 | 4.74 | 0.175 | 9064.73 | 3874 | 1.13 | 3.77 |
| 2011 | JiangXi | 164 | 544 | 55 | 2.62 | 0.148 | 3249.87 | 3289 | 2.06 | 6.40 |
| 2011 | ShanDong | 801 | 3829 | 53 | 4.73 | 0.120 | 9493.54 | 8914 | 1.35 | 15.45 |
| 2011 | HeNan | 549 | 1789 | 57 | 2.87 | 0.150 | 2804.39 | 6234 | 0.52 | 9.92 |
| 2011 | HuBei | 374 | 1627 | 50 | 3.42 | 0.117 | 3436.27 | 4309 | 1.32 | 4.23 |
| 2011 | HuNan | 286 | 1616 | 48 | 2.99 | 0.117 | 2314.77 | 4470 | 0.65 | 6.22 |
| 2011 | GuangDong | 521 | 7331 | 50 | 5.08 | 0.190 | 29960.03 | 10119 | 0.62 | 9.51 |
| 2011 | GuangXi | 192 | 575 | 48 | 2.53 | 0.159 | 1982.62 | 3342 | 1.38 | 3.12 |
| 2011 | HaiNan | 35 | 98 | 28 | 2.89 | 0.142 | 1461.12 | 687 | 1.11 | 0.35 |
| 2011 | ChongQing | 160 | 1191 | 55 | 3.45 | 0.100 | 2992.50 | 874 | 2.59 | 3.88 |
| 2011 | SiChuan | 303 | 1670 | 53 | 2.61 | 0.122 | 3802.02 | 5721 | 0.52 | 6.22 |
| 2011 | GuiZhou | 211 | 264 | 39 | 1.64 | 0.128 | 376.20 | 1080 | 1.14 | 4.47 |
| 2011 | YunNan | 205 | 702 | 43 | 1.93 | 0.155 | 1366.75 | 1500 | 1.34 | 3.16 |
| 2011 | ShanXi | 244 | 1347 | 55 | 3.35 | 0.124 | 1316.86 | 3110 | 0.61 | 5.25 |
| 2011 | GanSu | 139 | 316 | 47 | 1.96 | 0.175 | 423.36 | 1362 | 1.19 | 2.64 |
| 2011 | QingHai | 37 | 33 | 58 | 2.95 | 0.216 | 208.15 | 368 | 1.57 | 0.70 |
| 2011 | NingXia | 137 | 84 | 50 | 3.30 | 0.206 | 291.30 | 571 | 2.73 | 2.02 |
| 2011 | XinJiang | 203 | 254 | 49 | 3.01 | 0.104 | 370.97 | 2278 | 2.01 | 4.10 |
| 2012 | BeiJing | 97 | 7472 | 23 | 8.75 | 0.156 | 9409.53 | 17613 | 1.92 | 0.34 |
| 2012 | TianJin | 158 | 1967 | 52 | 9.32 | 0.115 | 7491.61 | 5299 | 1.22 | 2.09 |
| 2012 | HeBei | 715 | 1580 | 53 | 3.66 | 0.125 | 3084.53 | 28941 | 1.83 | 16.95 |
| 2012 | ShanXi | 466 | 539 | 56 | 3.36 | 0.112 | 2013.70 | 18597 | 2.71 | 13.66 |
| 2012 | NeiMengGu | 622 | 437 | 55 | 6.39 | 0.125 | 1625.56 | 9205 | 2.80 | 12.44 |
| 2012 | LiaoNing | 461 | 2209 | 53 | 5.66 | 0.099 | 11690.70 | 23800 | 2.75 | 14.07 |
| 2012 | JiLin | 230 | 568 | 53 | 4.34 | 0.102 | 1505.08 | 10107 | 0.87 | 3.64 |
| 2012 | HeiLongJiang | 269 | 925 | 44 | 3.57 | 0.080 | 1401.57 | 14206 | 1.59 | 4.73 |
| 2012 | ShangHai | 195 | 4398 | 39 | 8.54 | 0.146 | 26067.78 | 10590 | 0.66 | 1.90 |
| 2012 | JiangSu | 656 | 11120 | 50 | 6.83 | 0.195 | 39375.62 | 44533 | 1.22 | 20.10 |
| 2012 | ZheJiang | 377 | 6489 | 50 | 6.34 | 0.218 | 13722.23 | 35719 | 1.08 | 9.52 |
| 2012 | AnHui | 318 | 2709 | 55 | 2.88 | 0.147 | 2517.63 | 26502 | 1.92 | 5.85 |
| 2012 | FuJian | 232 | 1680 | 52 | 5.28 | 0.174 | 9182.04 | 18726 | 1.13 | 3.34 |
| 2012 | JiangXi | 164 | 642 | 54 | 2.88 | 0.147 | 3393.05 | 15192 | 2.44 | 8.43 |
| 2012 | ShanDong | 842 | 4756 | 51 | 5.18 | 0.120 | 9961.35 | 42267 | 1.48 | 16.18 |
| 2012 | HeNan | 521 | 1687 | 56 | 3.15 | 0.152 | 2919.56 | 30248 | 0.71 | 11.18 |
| 2012 | HuBei | 368 | 1974 | 50 | 3.86 | 0.114 | 3671.31 | 19611 | 1.28 | 4.21 |
| 2012 | HuNan | 282 | 1919 | 47 | 3.35 | 0.116 | 2418.02 | 21027 | 0.86 | 6.20 |
| 2012 | GuangDong | 505 | 7665 | 49 | 5.41 | 0.195 | 30155.13 | 46114 | 0.46 | 9.01 |
| 2012 | GuangXi | 205 | 1036 | 48 | 2.80 | 0.155 | 1962.03 | 14904 | 1.46 | 3.17 |
| 2012 | HaiNan | 37 | 115 | 28 | 3.24 | 0.153 | 1705.56 | 2911 | 1.57 | 0.36 |
| 2012 | ChongQing | 165 | 1144 | 52 | 3.89 | 0.096 | 3382.76 | 4401 | 1.64 | 3.79 |
| 2012 | SiChuan | 331 | 2688 | 52 | 2.96 | 0.120 | 4034.92 | 23348 | 0.75 | 5.96 |
| 2012 | GuiZhou | 230 | 406 | 39 | 1.97 | 0.130 | 483.20 | 4909 | 1.01 | 5.17 |
| 2012 | YunNan | 212 | 853 | 43 | 2.22 | 0.155 | 1421.37 | 6150 | 1.28 | 3.64 |
| 2012 | ShanXi | 262 | 1893 | 56 | 3.86 | 0.123 | 1961.20 | 14087 | 1.25 | 5.60 |
| 2012 | GanSu | 153 | 437 | 46 | 2.20 | 0.174 | 439.67 | 6012 | 2.15 | 2.40 |
| 2012 | QingHai | 45 | 88 | 58 | 3.32 | 0.210 | 178.24 | 2107 | 1.27 | 0.73 |
| 2012 | NingXia | 135 | 181 | 50 | 3.64 | 0.200 | 195.19 | 2502 | 2.38 | 2.18 |
| 2012 | XinJiang | 252 | 286 | 46 | 3.38 | 0.120 | 419.26 | 4154 | 3.40 | 4.67 |
| 2013 | BeiJing | 93 | 8423 | 22 | 9.46 | 0.166 | 11139.06 | 81175 | 2.22 | 0.33 |
| 2013 | TianJin | 157 | 2094 | 51 | 10.01 | 0.124 | 8013.08 | 18720 | 1.33 | 1.88 |
| 2013 | HeBei | 769 | 1547 | 52 | 3.89 | 0.135 | 3427.89 | 70805 | 1.73 | 16.67 |
| 2013 | ShanXi | 488 | 628 | 54 | 3.50 | 0.114 | 2151.08 | 36035 | 2.68 | 16.05 |
| 2013 | NeiMengGu | 576 | 409 | 54 | 6.78 | 0.152 | 1440.34 | 18860 | 3.01 | 13.04 |
| 2013 | LiaoNing | 482 | 2347 | 53 | 6.20 | 0.114 | 11522.73 | 44249 | 1.28 | 15.15 |
| 2013 | JiLin | 222 | 662 | 53 | 4.74 | 0.094 | 2000.12 | 19731 | 0.81 | 3.51 |
| 2013 | HeiLongJiang | 257 | 1075 | 41 | 3.77 | 0.087 | 1434.05 | 25654 | 2.08 | 4.98 |
| 2013 | ShangHai | 201 | 4261 | 37 | 9.10 | 0.153 | 28800.54 | 54435 | 0.87 | 2.25 |
| 2013 | JiangSu | 694 | 11421 | 49 | 7.54 | 0.205 | 41914.56 | 100922 | 1.49 | 20.43 |
| 2013 | ZheJiang | 379 | 6889 | 49 | 6.88 | 0.228 | 15120.44 | 78328 | 1.04 | 8.84 |
| 2013 | AnHui | 343 | 3228 | 55 | 3.20 | 0.161 | 2616.52 | 49543 | 2.66 | 5.60 |
| 2013 | FuJian | 229 | 1678 | 52 | 5.81 | 0.195 | 9843.38 | 36044 | 1.30 | 3.50 |
| 2013 | JiangXi | 197 | 670 | 54 | 3.19 | 0.154 | 3698.34 | 25926 | 1.67 | 8.45 |
| 2013 | ShanDong | 762 | 5796 | 50 | 5.69 | 0.142 | 11101.32 | 78177 | 1.55 | 16.71 |
| 2013 | HeNan | 484 | 1892 | 55 | 3.42 | 0.173 | 3006.48 | 60768 | 0.90 | 9.86 |
| 2013 | HuBei | 309 | 2076 | 49 | 4.28 | 0.142 | 4113.46 | 42601 | 1.02 | 4.33 |
| 2013 | HuNan | 271 | 2067 | 47 | 3.69 | 0.124 | 2547.33 | 40012 | 0.95 | 5.81 |
| 2013 | GuangDong | 497 | 7957 | 47 | 5.88 | 0.208 | 32241.00 | 92412 | 0.57 | 9.56 |
| 2013 | GuangXi | 210 | 1508 | 48 | 3.07 | 0.167 | 2006.41 | 30349 | 1.52 | 3.28 |
| 2013 | HaiNan | 40 | 87 | 28 | 3.57 | 0.166 | 1698.22 | 7099 | 0.85 | 0.45 |
| 2013 | ChongQing | 140 | 1256 | 51 | 4.32 | 0.124 | 3698.34 | 10041 | 1.37 | 3.99 |
| 2013 | SiChuan | 343 | 2804 | 52 | 3.26 | 0.127 | 4560.03 | 50811 | 0.89 | 7.21 |
| 2013 | GuiZhou | 233 | 485 | 41 | 2.32 | 0.149 | 748.47 | 11180 | 1.37 | 5.32 |
| 2013 | YunNan | 206 | 864 | 42 | 2.53 | 0.159 | 1515.82 | 12131 | 1.68 | 3.58 |
| 2013 | ShanXi | 266 | 2067 | 56 | 4.31 | 0.133 | 2302.03 | 34348 | 1.38 | 6.22 |
| 2013 | GanSu | 160 | 541 | 45 | 2.45 | 0.181 | 408.83 | 11543 | 2.81 | 2.34 |
| 2013 | QingHai | 48 | 113 | 57 | 3.69 | 0.221 | 188.69 | 5382 | 1.75 | 0.77 |
| 2013 | NingXia | 143 | 242 | 49 | 3.96 | 0.204 | 220.14 | 5459 | 2.82 | 2.00 |
| 2013 | XinJiang | 293 | 358 | 45 | 3.76 | 0.144 | 408.83 | 8273 | 3.81 | 6.34 |
| 2014 | BeiJing | 93 | 8752 | 21 | 10.00 | 0.168 | 12347.03 | 171214 | 2.93 | 2.78 |
| 2014 | TianJin | 155 | 2107 | 49 | 10.52 | 0.124 | 8851.77 | 32373 | 1.77 | 3.81 |
| 2014 | HeBei | 752 | 1485 | 51 | 4.00 | 0.139 | 3814.68 | 88843 | 1.55 | 15.40 |
| 2014 | ShanXi | 476 | 704 | 49 | 3.51 | 0.113 | 2401.83 | 44765 | 2.30 | 12.12 |
| 2014 | NeiMengGu | 582 | 406 | 51 | 7.10 | 0.162 | 1621.70 | 21566 | 3.16 | 10.49 |
| 2014 | LiaoNing | 485 | 2142 | 50 | 6.52 | 0.115 | 12199.60 | 56504 | 0.95 | 13.27 |
| 2014 | JiLin | 223 | 574 | 53 | 5.02 | 0.096 | 2045.55 | 23107 | 0.71 | 3.33 |
| 2014 | HeiLongJiang | 269 | 1235 | 37 | 3.92 | 0.086 | 1474.27 | 31481 | 1.21 | 3.78 |
| 2014 | ShangHai | 188 | 3949 | 35 | 9.74 | 0.152 | 32587.55 | 58658 | 1.06 | 1.77 |
| 2014 | JiangSu | 705 | 10995 | 47 | 8.19 | 0.206 | 44111.45 | 106342 | 1.35 | 18.76 |
| 2014 | ZheJiang | 375 | 5876 | 48 | 7.30 | 0.229 | 16149.42 | 86559 | 1.18 | 10.09 |
| 2014 | AnHui | 350 | 4557 | 53 | 3.44 | 0.162 | 2948.54 | 55188 | 2.06 | 5.75 |
| 2014 | FuJian | 243 | 1573 | 52 | 6.35 | 0.189 | 10639.33 | 39348 | 0.80 | 3.31 |
| 2014 | JiangXi | 202 | 863 | 53 | 3.47 | 0.155 | 4115.68 | 31016 | 1.47 | 8.58 |
| 2014 | ShanDong | 790 | 5421 | 48 | 6.09 | 0.142 | 12236.46 | 102077 | 1.39 | 14.87 |
| 2014 | HeNan | 535 | 1987 | 51 | 3.71 | 0.170 | 3618.11 | 74482 | 0.84 | 8.22 |
| 2014 | HuBei | 310 | 2278 | 47 | 4.71 | 0.140 | 4772.96 | 46853 | 1.16 | 4.22 |
| 2014 | HuNan | 270 | 2320 | 46 | 4.03 | 0.121 | 2844.12 | 44570 | 0.79 | 5.69 |
| 2014 | GuangDong | 504 | 7204 | 46 | 6.35 | 0.217 | 34528.68 | 116739 | 0.45 | 8.78 |
| 2014 | GuangXi | 208 | 1830 | 47 | 3.31 | 0.169 | 2297.41 | 33619 | 1.28 | 2.48 |
| 2014 | HaiNan | 41 | 99 | 25 | 3.89 | 0.170 | 1713.84 | 8672 | 0.60 | 0.44 |
| 2014 | ChongQing | 156 | 1245 | 46 | 4.79 | 0.124 | 4146.39 | 12048 | 1.18 | 3.68 |
| 2014 | SiChuan | 341 | 3014 | 49 | 3.51 | 0.127 | 5086.24 | 57630 | 1.07 | 5.67 |
| 2014 | GuiZhou | 231 | 683 | 42 | 2.64 | 0.149 | 952.13 | 12622 | 1.84 | 4.20 |
| 2014 | YunNan | 195 | 1060 | 41 | 2.73 | 0.180 | 1554.13 | 13705 | 1.19 | 3.47 |
| 2014 | ShanXi | 277 | 1871 | 54 | 4.69 | 0.134 | 2745.83 | 37398 | 1.61 | 6.33 |
| 2014 | GanSu | 164 | 652 | 43 | 2.64 | 0.179 | 417.71 | 13254 | 2.10 | 2.03 |
| 2014 | QingHai | 49 | 102 | 54 | 3.97 | 0.223 | 190.43 | 6763 | 1.30 | 0.74 |
| 2014 | NingXia | 143 | 286 | 49 | 4.18 | 0.211 | 319.43 | 6269 | 2.86 | 2.88 |
| 2014 | XinJiang | 329 | 469 | 43 | 4.06 | 0.158 | 466.85 | 9355 | 4.24 | 6.85 |
| 2015 | BeiJing | 92 | 9326 | 20 | 10.65 | 0.171 | 23727.87 | 111052 | 1.79 | 3.80 |
| 2015 | TianJin | 152 | 3099 | 47 | 10.80 | 0.127 | 11293.83 | 22925 | 0.76 | 6.38 |
| 2015 | HeBei | 734 | 2054 | 48 | 4.03 | 0.133 | 4585.61 | 78322 | 1.33 | 17.40 |
| 2015 | ShanXi | 440 | 751 | 41 | 3.49 | 0.110 | 2560.30 | 44901 | 2.02 | 11.65 |
| 2015 | NeiMengGu | 585 | 549 | 50 | 7.11 | 0.165 | 2188.79 | 24826 | 3.01 | 10.25 |
| 2015 | LiaoNing | 472 | 2302 | 45 | 6.54 | 0.113 | 12870.33 | 58633 | 1.02 | 11.33 |
| 2015 | JiLin | 208 | 750 | 50 | 5.11 | 0.098 | 2194.28 | 27305 | 0.79 | 3.80 |
| 2015 | HeiLongJiang | 265 | 1487 | 32 | 3.95 | 0.088 | 1389.06 | 29414 | 1.04 | 4.36 |
| 2015 | ShangHai | 189 | 4650 | 32 | 10.38 | 0.152 | 41186.76 | 75078 | 0.88 | 2.69 |
| 2015 | JiangSu | 704 | 12685 | 46 | 8.80 | 0.208 | 48715.66 | 107730 | 1.36 | 22.18 |
| 2015 | ZheJiang | 375 | 7726 | 46 | 7.76 | 0.223 | 18175.31 | 81070 | 1.03 | 9.31 |
| 2015 | AnHui | 351 | 5953 | 50 | 3.60 | 0.163 | 6632.40 | 63440 | 2.00 | 5.89 |
| 2015 | FuJian | 230 | 2331 | 50 | 6.80 | 0.190 | 12252.06 | 38196 | 0.88 | 3.99 |
| 2015 | JiangXi | 210 | 1222 | 50 | 3.67 | 0.158 | 4520.46 | 31391 | 1.41 | 8.50 |
| 2015 | ShanDong | 825 | 6957 | 47 | 6.42 | 0.168 | 13660.97 | 97943 | 1.10 | 11.35 |
| 2015 | HeNan | 518 | 2593 | 48 | 3.91 | 0.172 | 4279.53 | 82277 | 1.00 | 8.28 |
| 2015 | HuBei | 308 | 2799 | 46 | 5.07 | 0.140 | 5557.68 | 45014 | 0.84 | 5.76 |
| 2015 | HuNan | 289 | 2828 | 44 | 4.28 | 0.122 | 3247.94 | 46352 | 1.86 | 5.25 |
| 2015 | GuangDong | 505 | 9429 | 45 | 6.75 | 0.217 | 40130.17 | 102125 | 0.40 | 7.50 |
| 2015 | GuangXi | 198 | 2209 | 46 | 3.52 | 0.168 | 2648.91 | 27282 | 1.55 | 2.61 |
| 2015 | HaiNan | 42 | 134 | 24 | 4.08 | 0.173 | 1941.61 | 8310 | 0.60 | 0.44 |
| 2015 | ChongQing | 159 | 2497 | 45 | 5.23 | 0.120 | 4910.76 | 13142 | 0.88 | 3.38 |
| 2015 | SiChuan | 323 | 4259 | 44 | 3.68 | 0.124 | 5506.49 | 61230 | 1.22 | 6.05 |
| 2015 | GuiZhou | 234 | 898 | 39 | 2.98 | 0.145 | 1130.26 | 13446 | 1.31 | 3.95 |
| 2015 | YunNan | 176 | 1213 | 40 | 2.88 | 0.171 | 2037.91 | 17620 | 1.03 | 2.55 |
| 2015 | ShanXi | 277 | 1766 | 50 | 4.76 | 0.128 | 3212.04 | 35200 | 1.33 | 6.46 |
| 2015 | GanSu | 159 | 619 | 37 | 2.62 | 0.179 | 476.91 | 14712 | 1.80 | 2.15 |
| 2015 | QingHai | 51 | 183 | 50 | 4.13 | 0.196 | 460.65 | 5548 | 1.44 | 0.78 |
| 2015 | NingXia | 141 | 232 | 47 | 4.38 | 0.200 | 558.83 | 6388 | 2.98 | 2.04 |
| 2015 | XinJiang | 344 | 543 | 39 | 4.00 | 0.172 | 530.56 | 8933 | 3.10 | 6.19 |
| 2016 | BeiJing | 89 | 10606 | 19 | 11.82 | 0.180 | 28387.24 | 134800 | 2.63 | 6.18 |
| 2016 | TianJin | 147 | 3636 | 42 | 11.51 | 0.128 | 14785.36 | 28818 | 0.30 | 6.81 |
| 2016 | HeBei | 748 | 2488 | 48 | 4.31 | 0.135 | 5634.08 | 83978 | 1.25 | 19.40 |
| 2016 | ShanXi | 449 | 987 | 39 | 3.55 | 0.114 | 2800.61 | 50682 | 4.03 | 11.18 |
| 2016 | NeiMengGu | 590 | 603 | 47 | 7.21 | 0.165 | 2728.66 | 25570 | 2.52 | 10.02 |
| 2016 | LiaoNing | 457 | 3025 | 39 | 5.08 | 0.122 | 14166.59 | 55379 | 0.79 | 12.07 |
| 2016 | JiLin | 201 | 975 | 47 | 5.39 | 0.102 | 2365.03 | 25590 | 0.57 | 3.94 |
| 2016 | HeiLongJiang | 269 | 1520 | 29 | 4.04 | 0.090 | 1878.47 | 33665 | 1.13 | 4.60 |
| 2016 | ShangHai | 188 | 5305 | 30 | 11.66 | 0.156 | 48770.85 | 58435 | 0.73 | 3.60 |
| 2016 | JiangSu | 724 | 17082 | 45 | 9.69 | 0.216 | 58443.48 | 102313 | 0.99 | 24.39 |
| 2016 | ZheJiang | 372 | 9899 | 45 | 8.49 | 0.235 | 21246.72 | 79507 | 1.38 | 8.58 |
| 2016 | AnHui | 362 | 8834 | 48 | 3.96 | 0.174 | 4467.36 | 50186 | 2.04 | 6.04 |
| 2016 | FuJian | 213 | 3603 | 49 | 7.47 | 0.199 | 15032.55 | 36126 | 0.66 | 4.66 |
| 2016 | JiangXi | 213 | 1818 | 48 | 4.04 | 0.166 | 5163.60 | 31008 | 1.69 | 8.20 |
| 2016 | ShanDong | 833 | 7862 | 46 | 6.87 | 0.176 | 16730.21 | 98329 | 1.15 | 17.09 |
| 2016 | HeNan | 513 | 3279 | 48 | 4.26 | 0.171 | 5463.21 | 96946 | 0.89 | 8.87 |
| 2016 | HuBei | 311 | 4277 | 45 | 5.57 | 0.144 | 6596.88 | 40920 | 1.42 | 7.29 |
| 2016 | HuNan | 294 | 3532 | 42 | 4.64 | 0.123 | 3852.53 | 42319 | 0.64 | 4.80 |
| 2016 | GuangDong | 518 | 13248 | 43 | 7.40 | 0.221 | 51914.28 | 92920 | 0.45 | 6.22 |
| 2016 | GuangXi | 211 | 4154 | 45 | 3.80 | 0.166 | 2904.03 | 27305 | 1.11 | 3.63 |
| 2016 | HaiNan | 40 | 148 | 22 | 4.43 | 0.176 | 5050.73 | 7805 | 0.75 | 0.55 |
| 2016 | ChongQing | 154 | 2108 | 45 | 5.85 | 0.123 | 5849.55 | 12941 | 0.81 | 3.08 |
| 2016 | SiChuan | 310 | 5411 | 41 | 4.00 | 0.127 | 6256.56 | 91189 | 1.43 | 6.27 |
| 2016 | GuiZhou | 249 | 1020 | 40 | 3.32 | 0.149 | 1575.51 | 12954 | 1.01 | 4.48 |
| 2016 | YunNan | 180 | 1633 | 38 | 3.11 | 0.163 | 2192.27 | 17565 | 0.99 | 2.54 |
| 2016 | ShanXi | 265 | 2503 | 49 | 5.10 | 0.148 | 3725.07 | 42987 | 1.64 | 6.59 |
| 2016 | GanSu | 152 | 995 | 35 | 2.76 | 0.178 | 500.09 | 17350 | 1.63 | 2.72 |
| 2016 | QingHai | 56 | 197 | 49 | 4.35 | 0.191 | 499.99 | 5609 | 2.19 | 0.82 |
| 2016 | NingXia | 137 | 396 | 47 | 4.72 | 0.195 | 578.34 | 7371 | 3.19 | 2.24 |
| 2016 | XinJiang | 370 | 644 | 38 | 4.06 | 0.178 | 642.04 | 8316 | 3.24 | 6.12 |
| 2017 | BeiJing | 85 | 8835 | 19 | 12.90 | 0.184 | 32841.34 | 134694 | 2.38 | 8.56 |
| 2017 | TianJin | 141 | 2859 | 41 | 11.89 | 0.131 | 17205.13 | 23303 | 0.38 | 8.23 |
| 2017 | HeBei | 726 | 2497 | 47 | 4.54 | 0.145 | 6469.45 | 89089 | 1.68 | 25.86 |
| 2017 | ShanXi | 488 | 1074 | 44 | 4.21 | 0.122 | 3357.30 | 52971 | 1.86 | 11.56 |
| 2017 | NeiMengGu | 639 | 644 | 40 | 6.38 | 0.178 | 3104.44 | 25513 | 2.61 | 9.78 |
| 2017 | LiaoNing | 479 | 2303 | 39 | 5.35 | 0.124 | 21325.56 | 58110 | 0.92 | 12.81 |
| 2017 | JiLin | 204 | 1001 | 47 | 5.48 | 0.108 | 2624.67 | 28226 | 0.60 | 3.29 |
| 2017 | HeiLongJiang | 269 | 1181 | 26 | 4.19 | 0.091 | 2273.24 | 38989 | 0.81 | 3.97 |
| 2017 | ShangHai | 190 | 4802 | 30 | 12.66 | 0.158 | 53895.50 | 51642 | 0.53 | 4.51 |
| 2017 | JiangSu | 736 | 17276 | 45 | 10.72 | 0.227 | 65210.15 | 108300 | 0.83 | 26.60 |
| 2017 | ZheJiang | 382 | 10431 | 43 | 9.21 | 0.245 | 25212.20 | 80932 | 0.87 | 7.85 |
| 2017 | AnHui | 371 | 8993 | 48 | 4.34 | 0.181 | 5849.84 | 61677 | 1.84 | 6.19 |
| 2017 | FuJian | 230 | 3749 | 48 | 8.27 | 0.205 | 17603.34 | 39168 | 0.69 | 5.34 |
| 2017 | JiangXi | 224 | 2175 | 48 | 4.34 | 0.177 | 5455.27 | 35614 | 1.52 | 8.44 |
| 2017 | ShanDong | 806 | 7055 | 45 | 7.28 | 0.182 | 20540.17 | 99491 | 1.31 | 21.42 |
| 2017 | HeNan | 494 | 3955 | 47 | 4.67 | 0.175 | 7058.18 | 103870 | 1.43 | 7.50 |
| 2017 | HuBei | 325 | 4115 | 44 | 6.02 | 0.146 | 7771.50 | 48746 | 1.19 | 8.83 |
| 2017 | HuNan | 310 | 3850 | 42 | 4.96 | 0.120 | 11031.90 | 52861 | 0.63 | 5.20 |
| 2017 | GuangDong | 542 | 14946 | 42 | 8.09 | 0.226 | 118982.06 | 109267 | 0.41 | 4.95 |
| 2017 | GuangXi | 221 | 3307 | 40 | 3.81 | 0.170 | 3794.53 | 35102 | 0.90 | 3.70 |
| 2017 | HaiNan | 42 | 214 | 22 | 4.84 | 0.178 | 5137.38 | 8781 | 1.21 | 4.46 |
| 2017 | ChongQing | 158 | 2058 | 44 | 6.34 | 0.128 | 6384.39 | 22194 | 1.14 | 2.77 |
| 2017 | SiChuan | 309 | 5661 | 39 | 4.47 | 0.130 | 7615.84 | 108275 | 1.50 | 7.53 |
| 2017 | GuiZhou | 255 | 1478 | 40 | 3.80 | 0.162 | 2110.03 | 15756 | 1.60 | 5.01 |
| 2017 | YunNan | 195 | 1610 | 38 | 3.42 | 0.170 | 2523.98 | 22271 | 0.86 | 3.83 |
| 2017 | ShanXi | 262 | 2481 | 50 | 5.73 | 0.155 | 5404.11 | 54071 | 1.42 | 6.71 |
| 2017 | GanSu | 151 | 989 | 34 | 2.85 | 0.190 | 1363.69 | 20370 | 1.16 | 3.29 |
| 2017 | QingHai | 53 | 228 | 44 | 4.40 | 0.201 | 519.84 | 6054 | 1.55 | 0.87 |
| 2017 | NingXia | 175 | 378 | 46 | 5.08 | 0.185 | 2053.91 | 7609 | 2.44 | 2.80 |
| 2017 | XinJiang | 404 | 675 | 40 | 4.49 | 0.182 | 899.53 | 9672 | 3.53 | 6.74 |
